# A Good Digestion

**Published:** 1876-01

**Authors:** 


### A GOOD DIGESTER.

A GOOD digestion is essential to good health. The necessary agent in digestion is called the *gastric juice.* That which, of all liquids known, is more nearly allied to the gastric juice, is vinegar; its action on the food, whether in the stomach or out of it, being very much alike. Hence it is that a little vinegar, or a pickle, removes indigestion in many cases. A very weak stomach will digest a handful of cold, raw cabbage, cut up fine, with a tablespoon of strong vinegar on it, about as soon as anything that can be eaten.

Dyspeptic persons had a thousand times better "top off" with a few teaspoons of strong vinegar, than with a plum pudding, or mince-pie, or a glass of wine, brandy, or champagne.

But, as a large amount of city sold vinegar is known to be nothing more than a mixture of oil of vitriol and water, it will be an economizer, as well as a health-preserver, if our wives would learn to make their own vinegar—having these two advantages, it would be a superior article, and its constituents would be known, to say nothing of its great economy.

The *Ohio Cultivator* advises thus:—Mix a gallon of molasses with a barrel of cider, warm it in a large kettle, then put the mixture in a barrel with a few sheets of brown paper. Keep it in a warm place with the bung open, through which a stick is inserted for stirring it, to break the scum and admit the air. The vinegar may be drawn as needed, and its place supplied by cider, which, in its turn, will be converted into vinegar. Or: In a well-glazed vessel, mix one-half gallon of luke-warm water with half a pint of New Orleans or West India molasses. Set in a store room in cool weather, and anywhere in warm weather. If the mixture is not disturbed, in six weeks it will form an excellent vinegar. After standing a few weeks it will form a mother, with the use of which any quantity of vinegar can be made in three weeks, by adding the mother to a mixture of warm water and molasses (in the same proportion as above), and allowing it to stand undisturbed for three weeks."

When the stomach is about to be distressed by an improper meal, nature sometimes excites the most earnest longings for an acid of some kind, and such persons should always have some good vinegar on hand, although tart fruits or grapes are a great deal better.

As for ourselves, we would be very clear of taking the vinegar, for we have a most unconquerable antipathy against swallowing anything as a medicine, we would rather suffer for the time being; but we would eat less at the next meal, and then not be bothered with the longing.

